# Mutations in the potassium channel subunit KCNE1 are associated with early-onset familial atrial fibrillation

**DOI:** 10.1186/1471-2350-13-24

**Published:** 2012-04-03

**Authors:** Morten S Olesen, Bo H Bentzen, Jonas B Nielsen, Annette B Steffensen, Jens-Peter David, Javad Jabbari, Henrik K Jensen, Stig Haunsø, Jesper H Svendsen, Nicole Schmitt

**Affiliations:** 1 The Danish National Research Foundation Centre for Cardiac Arrhythmia, Copenhagen, Denmark; 2 Laboratory for Molecular Cardiology, The Heart Centre, Rigshospitalet, University of Copenhagen, Copenhagen, Denmark; 3 The Ion Channel Group, Department of Biomedical Sciences, Faculty of Health Sciences, University of Copenhagen, Blegdamsvej 3, 12.5.14, Copenhagen, N 2200, Denmark; 4 Department of Cardiology, Aarhus University Hospital, Skejby, Denmark; 5 Department of Surgery and Medicine, Faculty of Health Sciences, University of Copenhagen, Copenhagen, Denmark

**Keywords:** Lone AF, Genetics, K_V_7.1, KCNE1, I_Ks _current

## Abstract

**Background:**

Atrial fibrillation (AF) is the most common arrhythmia. The potassium current I_Ks _is essential for cardiac repolarization. Gain-of-function mutations in K_V_7.1, the pore-forming α-subunit of the I_Ks _channel, have been associated with AF. We hypothesized that early-onset lone AF is associated with mutations in the I_Ks _channel regulatory subunit KCNE1.

**Methods:**

In 209 unrelated early-onset lone AF patients (< 40 years) the entire coding sequence of *KCNE1 *was bidirectionally sequenced. We analyzed the identified KCNE1 mutants electrophysiologically in heterologous expression systems.

**Results:**

Two non-synonymous mutations G25V and G60D were found in *KCNE1 *that were not present in the control group (n = 432 alleles) and that have not previously been reported in any publicly available databases or in the exom variant server holding exom data from more than 10.000 alleles. Proband 1 (female, age 45, G25V) had onset of paroxysmal AF at the age of 39 years. Proband 2 (G60D) was diagnosed with lone AF at the age of 33 years. The patient has inherited the mutation from his mother, who also has AF. Both probands had no mutations in genes previously associated with AF. In heterologous expression systems, both mutants showed significant gain-of-function for I_Ks _both with respect to steady-state current levels, kinetic parameters, and heart rate-dependent modulation.

**Conclusions:**

Mutations in K_V_7.1 leading to gain-of-function of I_Ks _current have previously been described in lone AF, yet this is the first time a mutation in the beta-subunit *KCNE1 *is associated with the disease. This finding further supports the hypothesis that increased potassium current enhances AF susceptibility.

## Background

Atrial fibrillation (AF) is the most prevalent sustained cardiac arrhythmia. It is responsible for considerable morbidity and mortality, and its population prevalence has reached epidemic proportions, affecting almost seven million patients in the European Union and the USA combined [1-4].

In most cases AF is associated with cardiac risk factors such as hypertensive, ischemic, and/or structural heart disease [1,5]. However, 10-20% of patients suffering from AF are younger than 60 years of age and lack the traditional risk factors for AF. These patients are considered as having "lone" AF [2]. The mechanisms underlying AF are not fully understood, but a heterogeneous model based on the interaction of multiple substrates and triggers is thought to underlie the pathophysiology of the disease. However, early-onset lone AF has been suggested to be a primary electrical disease caused by disturbances in ionic currents. Of note, a genetic cause of these types of electrical disturbances is becoming increasingly recognized [6].

Identification of the genetic components of AF, and the importance of single nucleotide polymorphisms (SNPs) was recently shown in genome-wide association studies indicating that common variants also play a role in the development of AF [7,8]. This association between SNPs and AF was strongest in patients diagnosed at a younger age. There is evidence that variations in genes encoding ion channel subunits are associated with familial predisposition for AF. Several genetic reports have revealed mutations associated with AF in cardiac ion channels and accessory subunits [6]. Most of these studies show that gain- or loss-of-function mutations in the genes encoding proteins contributing to cardiac depolarization, e.g. *SCN1-3B *(involved in I_Na_) [6,9], or cardiac repolarisation, e.g. *KCNQ1 *(I_Ks_), *KCNH2 *(I_Kr_), *KCNJ2 *(I_K1_) can lead to increased susceptibility to AF [6]. These results support the two current conceptual models for AF. The first one being that cardiac action potential shortening functions as a substrate for re-entry wavelets in the atria [10,11] the second one proposing that a prolonged effective refractory period enhances the propensity for early after depolarization, and thereby increasing the susceptibility to AF [12].

K_V_7.1, the α-subunit of the I_Ks _current, has repeatedly been associated with AF [6]. Co-expression of its regulatory β-subunit KCNE1 changes the biophysical properties of the K_V_7.1 channel dramatically [13]. We hypothesized that early-onset lone AF is associated with mutations in *KCNE1*.

## Methods

An expanded Methods section is available in Additional file 1.

### Study subjects

Consecutive patients with lone AF and onset of AF before 40 years (i.e. absence of clinical or echocardiographic findings of other cardiovascular diseases, hypertension, metabolic or pulmonary diseases) were included from eight hospitals in the Copenhagen region of Denmark [9]. Healthy controls (216) were recruited from blood donors. The study conforms to the principles outlined in the Declaration of Helsinki and was approved by the Scientific Ethics Committee of Copenhagen and Frederiksberg (Protocol reference number KF 01313322). All included patients gave written informed consent.

### Mutation screening

Genomic DNA was extracted from blood samples using the QIAamp DNA Blood Mini Kit (QIAGEN, Hilden, Germany). Oligonucleotide primers for exons and splice junctions were designed using the known sequence of human KCNE1 [Genbank:NG_009091.1]. All primers were designed with M13 tail sequences. DNA fragments amplified by Touchdown PCR were analyzed using a high-resolution melting curve analysis (Light Scanner, Idaho Technology, UT, USA). Fragments with melting curves differing from the curves of wild-type DNA were purified and directly sequenced using M13 primers and Big Dye chemistry (DNA analyzer 3730, Applied Biosystems, CA, USA). The identified variants were validated by the resequencing of a second PCR product.

A group of 216 ethnically matched healthy controls was screened employing high resolution melting curve analysis (Light Scanner, Idaho technology, Salt Lake City, USA), and bidirectional sequencing of genes previously associated with AF was performed (Additional file 1). A mutation was considered suspected being disease causing if criteria previously defined were met [9].

### Molecular biology

Site-directed mutagenesis introducing the mutations G25V (c.74 C > T) and G60D (c.179 G > A) into human KCNE1 cDNA [Genbank:NM_000219.3] and *in vitro *transcription were performed using standard procedures. For a detailed description please refer to the Additional file 1.

### Heterologous expression studies

We employed two-electrode voltage-clamp experiments using *Xenopus laevis *oocytes expressing wild-type or mutant I_Ks _and patch-clamp experiments using mammalian cells. A detailed description is available in Additional file 1.

### Data analysis

Data analysis was performed with Igor Pro (Wavemetrics, Lake Oswego, OR, USA) and GraphPad Prism (GraphPad Software Inc., San Diego, CA, USA). I/V-curves were constructed by measuring the current at the end of a voltage-step to potentials ranging from -100 to +60 mV for TEVC recordings or +40 mV for patch-clamp experiments, respectively. The data was plotted against the corresponding membrane potentials. Similarly, peak tail-currents, measured at -120 mV for TEVC or -40 mV for patch-clamping experiments following the depolarizing step, were plotted against the membrane potential of the depolarizing step to construct the activation curves. A Boltzmann function (*I*/*I_max _*= *I_min _*+ (*I_max_*-*I_min_*)/(1 + exp((V_50_-V)/k))) was fitted to the activation curves to obtain the potential of half-maximal activation (V_50_) and the slope factor (k). Activation of K_V_7.1/KCNE1 channels results in sigmoidal activation current traces. In order to compare the activation kinetics we determined the time needed to reach the half-maximal current level (t_1/2_). An estimate of the values of the time constants of channel deactivation (τ) was obtained by fitting a mono-exponential function to tail-current traces measured at -140 to -40 mV following a voltage-step to +40 mV for TEVC or 0 mV for patch-clamp experiments.

The frequency dependence of conduction was investigated using three different voltage protocols (60, 120, and 180 bpm). The amount of charge conducted by the I_Ks _channel complex was calculated by integrating the area under the curve in the first 130 ms after the capacitive spike (10 ms) of the pulse at the 7th second. At this time point the current amplitude had reached a steady-state for all pacing frequencies tested. The amount of charge carried was normalized to the charge carried at 60 bpm.

Data are represented as mean ± SEM, unless otherwise indicated. Unpaired t-tests or ANOVA followed by Tukey's method of multiple comparisons were used as appropriate to compare the wild-type and mutated I_Ks _channel complex. P-values below 0.05 were considered statistically significant.

A detailed description of all methods used is available in Additional file 1.

## Results

### Study cohort

The study population consisted of 209 patients with onset of AF ranging from 16 to 39 years, without any concomitant disease. A control population of 216 healthy blood donors was collected (52% male gender, median age of 39 years (interquartile range 30-48 years)). Healthy controls were included after clinical evaluation and ECG recording. All included individuals were of Danish/Caucasian ethnicity. Clinical data of the study population is shown in Table 1[14].

**Table 1:** Clinical characteristics of the lone AF population (n = 209)

Median age of onset, y (IQR)	31.5 (26-36)
Male gender, %	82
Height, cm	183 ± 9
Weight, kg	89 ± 17
BMI, kg/m^2^	26.7 ± 4.6
Blood Pressure, mmHg	
Systolic	131 ± 13
Diastolic	78 ± 9
AF type	
Paroxysmal, %	55.9
Persistent, %	35.9
Permanent, %	8.2
Family history of AF	
1^st ^degree relatives with AF, %	31

All numbers are reported as mean ± standard-deviation unless otherwise noted. *IQR *Interquartile range.

### Mutation screening

**Figure 1:**
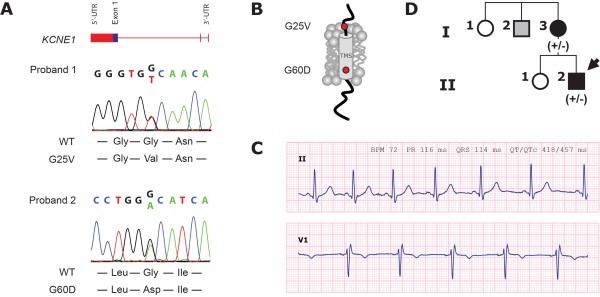
**Clinical characterization and genetic analysis of probands**. **A**: DNA sequence analysis. **B**: Positions of mutations indicated in schematic of protein topology. **C**: ECG from proband 2 (paper speed 25 mm/s, 1 mV/mm). **D**: Pedigree of the family with the novel *KCNE1 *G60D mutation. Squares: male, circles: female family members, respectively. Arrow indicates the proband 2. Solid black symbols indicate the presence of AF, open symbols: unaffected members, gray: AF history; (+/-): presence of the heterozygous mutation for persons with DNA samples available for testing.

Direct DNA sequencing of *KCNE1 *from the 209 index patients revealed two non-synonymous mutations in *KCNE1 *(c.74 g > t, G25V; c.179 G > A, G60D) (Figure 1). The mutations were not present in the control group (n = 216), and have not previously been reported to be associated with AF. All genetically affected probands were heterozygous carriers. The positions G25 and G60 in *KCNE1 *are highly conserved across species suggesting a functional importance (Additional file 1: Figure S1).

### Clinical data

Proband 1 (female, age 45) was diagnosed with paroxysmal AF at the age of 39 years. The sinus rhythm ECG showed an incomplete right bundle branch block (IRBBB; defined as an extra r'-wave in lead V1 and/or V2 [15]) but was otherwise normal (QT_c _401 ms) (data not shown). She had a normal echocardiography, and there was no history of AF in her family.

Proband 2 (male, age 45) had experienced distinct palpitations since the age of 20 years and AF was documented by ECG at the age of 33 years. At inclusion, he experienced weekly palpitations and chest discomfort lasting from one hour to half a day. The frequency of AF was higher during periods of physiological stress. The patient had a sinus rhythm ECG with IRBBB and a borderline long QT_c _interval where borderline prolonged in males is defined as QT_c _440-460 ms [16] (Figure 1C: P-wave 80 ms, PR 116 ms, QRS 114 ms, QT_c _457 ms). There was no sign of hypertrophy or ischemia in the ECG and echocardiography was normal. He never experienced syncope or near-syncope. For clinical purpose, the patient had an implantable loop recorder inserted for one year. The device revealed that the patient had AF 6% of the time and no ventricular arrhythmias were detected during the observation period. The patient's mother (I-3, Figure 1D) was diagnosed with paroxysmal AF and had palpitations since the age of 28 years. She also had a borderline long QT_c _interval of 459 ms and carries the mutation G60D in *KCNE1*. The proband's uncle (I-2) has a history of palpitations and chest discomfort, but was unavailable for genetic screening.

Both index patients were free of mutations in genes previously associated with AF (*KCNQ1*, *KCNH2, KCNN3*, *KCNA5*, *KCNE2/3/5*, *KCNJ2,5*, *SCN5A*, *SCN1-3B*, *ANP*, and *LMNA*).

#### KCNE1-G25V and KCNE1-G60D cause gain-of-function of I_Ks_

The slow delayed rectifier potassium current I_Ks _is important for terminating the cardiac action potential and is composed of K_V_7.1 and KCNE1 [17,18]. To examine whether a mutation in the accessory β-subunit KCNE1 could explain the lone AF phenotype observed in the proband, we expressed wild-type (WT) or mutant KCNE1 together with the pore forming K_V_7.1 α-subunit in *X.laevis *oocytes and recorded currents using the two-electrode voltage-clamp technique.

**Figure 2:**
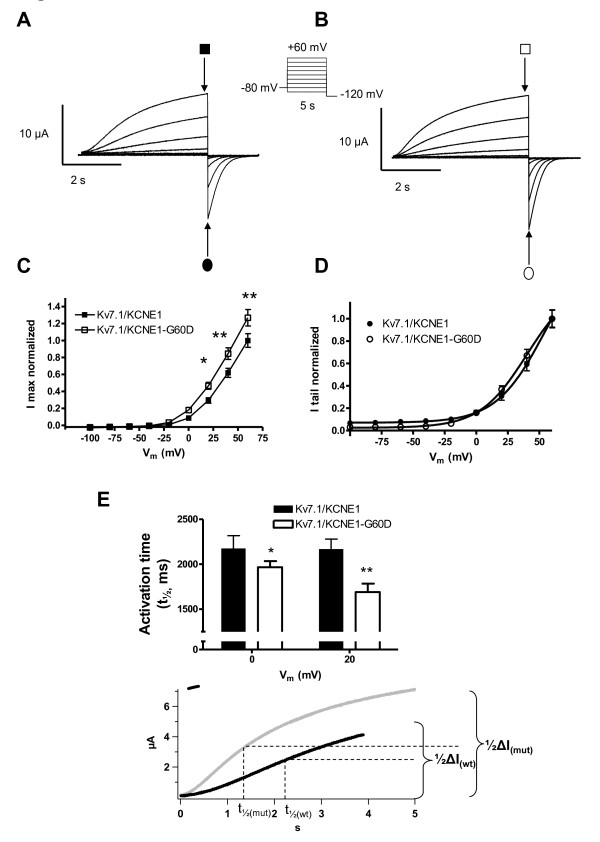
**Comparison of I_Ks_-WT and I_Ks_-G60D channel currents**. Representative current traces for KCNE1-WT (**A**) and KCNE1-G60D (**B**) channel subunits co-expressed with K_V_7.1 in *X.laevis *oocytes in a 1:1 molar ratio. Current step protocol is shown as inset. **C**: I/V relationship for I_Ks_-WT (n = 21) and I_Ks_-G60D (n = 23) as determined from peak currents (open and filled squares). **D**: Voltage-dependence of I_Ks_-WT and I_Ks_-G60D channel activation as determined from tail currents (open and filled circles). **E**: Activation rise time, determined as the time to 1/2 max following a depolarization to 0 mV or +20 mV.

Expression of K_V_7.1 and KCNE1 gave rise to a slowly activating and deactivating potassium current, that does not inactive (Figure 2A). Co-expression of the mutants KCNE1-G25V or KCNE1-G60D and K_V_7.1 (Figure 2B,Additional file 1: Figure S2) resulted in larger current amplitudes compared to the WT complex.

When summarizing the current measurements from oocytes expressing either KCNE1-WT, KCNE-G25V or KCNE-G60D together with K_V_7.1, subjected to 5 s depolarizing potentials from a holding potential of -80 mV we found that both mutations caused an increase in steady-state current amplitude at all activating potentials (Figure 2C, Additional file 1: Figure S2). Voltage-dependence of channel activation determined by tail-current analysis did not reveal any difference between KCNE1-WT and mutants (Figure 2D, Additional file 1: Figure S2).

The KCNE1 mutations also altered biophysical properties as visible from the current traces in Figure 2. Activation of K_V_7.1/KCNE1 channels results in sigmoidal activation current traces [19]. To compare the activation kinetics we determined the time needed to reach the half-maximal current level (t_1/2_) when currents were elicited at physiologically relevant potentials 0 and +20 mV. The activation was significantly faster for the mutated channels as compared with wild-type channels (Figure 2E, Additional file 1: Figure S2).

**Figure 3:**
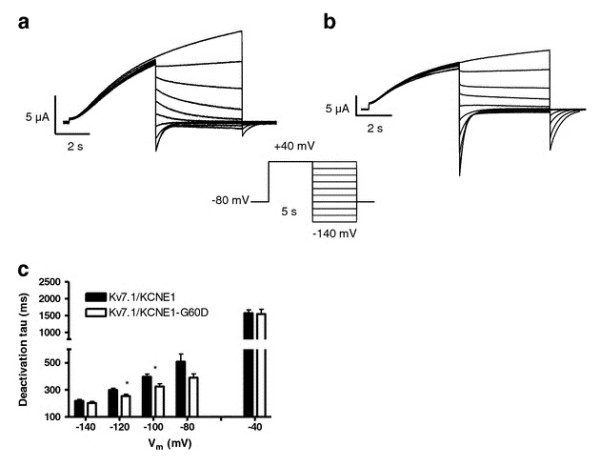
**Comparison of I_Ks_-WT and I_Ks_-G60D channel deactivation**. KCNE1-WT (**A**) and KCNE1-G60D (**B**) channel subunits co-expressed with K_V_7.1 in *X.laevis *oocytes in a 1:1 molar ratio. Current step protocol is shown as inset. **(C) **Enlargement of the tail-currents normalized to maximum current amplitude (gray: I_Ks_-WT; black: I_Ks_-G60D). Deactivation time constants (tau) were obtained by fitting the tail-current traces to a mono-exponential function **(D)**.

Deactivation kinetics were investigated by recording tail-currents at potentials ranging from -140 to -40 in 20 mV increments after an activating step to +40 mV (Figure 3A, B). The deactivating current traces were best fitted to a single exponential function. The summarized data in Figure 3C shows that expression of KCNE1-G60D resulted in a significantly faster deactivation when investigated at -100 and -120 mV. For KCNE1-G25V, deactivation kinetics were similar to WT (data not shown).

**Figure 4:**
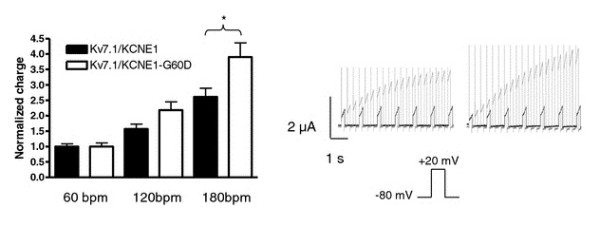
**Frequency dependence of conduction**. Three different voltage protocols were applied (60 bpm (+20 mV for 200 ms, followed by 800 ms at -80 mV); 120 bpm (+20 mV for 180 ms, followed by 320 ms at -80 mV); 180 bpm (+20 mV for 150 ms, followed by 180 ms at -80 mV)). The bar graph summarizes the amount of charge conducted by K_V_7.1-WT/KCNE1 (black; n = 10) or K_V_7.1/KCNE1-G60D (white; n = 15) channels in the first 130 ms after the capacitive spike (10 ms) of the pulse at the 7th second when normalized to the charge carried at 60 bpm. Representative current traces recorded (left: K_V_7.1/KCNE1; right: K_V_7.1/KCNE1-G60D) using the 60 bpm (black) and 120 bpm (gray) protocols are shown on the right.

Faster activation kinetics suggests a gain-of-function in I_Ks _whereas the opposite is true for the observed changes in deactivation kinetics. In the following, we focused on the functional characterization of G60D due to the more complex electrophysiological phenotype and due to the history of AF in this family. To address whether the changed channel kinetics of the mutant would result in altered heart rate-dependent modulation of I_Ks_, protocols mimicking heart rates of 60, 120, and 180 bpm were used. When *X.laevis *oocytes were subjected to voltage protocols at 60 bpm no accumulation of I_Ks _current was observed. In comparison, "stimulation" at 120 and 180 bpm resulted in an accumulation of I_Ks _current which reached significance for 180 bpm (see Figure 4). The amount of charge conducted by K_V_7.1/KCNE1-WT (n = 10) or K_V_7.1/KCNE1-G60D (n = 15) channels in the first 130 ms after the capacitive spike of the pulse at the 7th second was calculated and normalized to the charge carried at 60 bpm (Figure 4). The frequency-dependent buildup of conducted charge was significantly larger in the mutated I_Ks _channel complex as compared to WT when measured at 180 bpm.

To mimic the heterozygote state of the affected subjects we co-expressed K_V_7.1 with KCNE1-WT and KCNE1-G60D. This resulted in an intermediate phenotype both with respect to current amplitudes and channel kinetics. Activation kinetics were not significantly different from K_V_7.1/KCNE1 channels, but deactivation kinetics were significantly faster for the heterozygote state as compared to wild-type channels when measured at -120 to -80 mV (Additional file 1: Figure S3).

**Figure 5:**
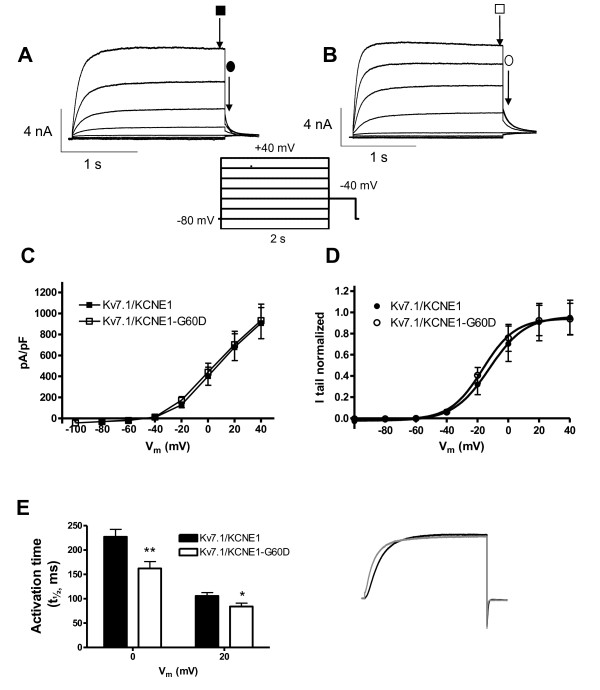
**Comparison of I_Ks_-WT and I_Ks_-G60D channel currents at 37°C**. KCNE1-WT (**A**) and KCNE1-G60D (**B**) channel subunits co-expressed with K_V_7.1 in CHO cells. Current protocol shown as inset. **C**: I/V relationship for I_Ks_-WT (n = 15) and I_Ks_-G60D (n = 12). Currents were measured at the end of the 2-second test pulse (open and filled squares) and normalized to cell size. **D**: Voltage-dependence of I_Ks_-WT and I_Ks_-G60D channel activation. Peak tail-currents were measured at -40 mV (open and filled circles), and the normalized data were fit to a two-state Boltzmann distribution. **E**: Activation rise time, determined as the time to 1/2 max following a depolarization to 0 or +20 mV.

**Figure 6:**
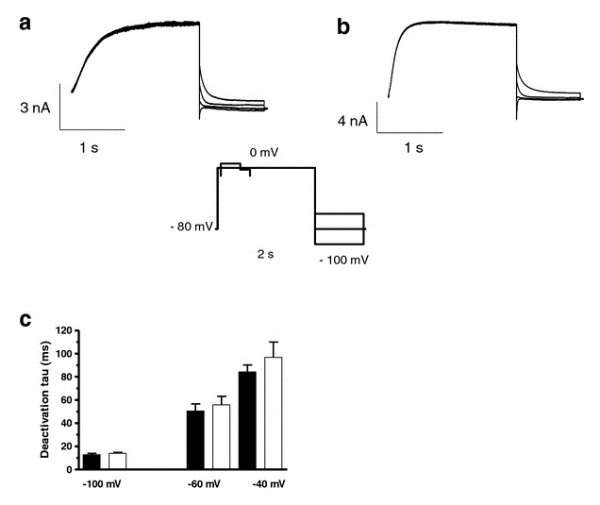
**Comparison of I_Ks_-WT and I_Ks_-G60D channel deactivation kinetics**. KCNE1-WT (**A**) and KCNE1-G60D (**B**) channel subunits co-expressed with K_V_7.1 in CHO cells. Currents were elicited by clamping the cells for 2 s at 0 mV, followed by a 1 s step to test potentials ranging from -100 to -20 mV in 20 mV increments, at 36 ± 1°C. **(C) **Enlargement of the tail-currents normalized to maximum current amplitude (gray: I_Ks_-WT; black: I_Ks_-G60D). Deactivation time constants (tau) were obtained by fitting the tail-current traces to a mono-exponential function **(D)**.

To verify the findings from the two-electrode voltage-clamp experiments, we performed whole cell patch-clamp experiments in CHO-K1 cells (Figure 5) at 36 ± 1°C. Under these conditions the expression of K_V_7.1 and KCNE1 resulted in a non-inactivating K_V_7.1/KCNE1 current with marked faster activation and deactivation kinetics as compared to when the channel subunits were expressed in *X.laevis *oocytes. In the mammalian expression system we observed no significant difference between the wild-type and mutated I_Ks _channel complex with respect to steady-state current amplitude or voltage-dependence of channel activation. However, the activation time was still significantly faster when the mutated β-subunit was expressed together with K_V_7.1. No significant difference in deactivation kinetics was observed between WT and mutant (Figure 6).

## Discussion

Although AF is the most common cardiac arrhythmia, the fundamental molecular pathways in many cases remain undefined. To our knowledge, the present study is the first to report that mutations in the β-subunit KCNE1 are associated with AF.

Both mutations (G25V, G60D) were absent in 216 matched controls, in publicly available databases and in 2276 exoms from the Popgen population NHLBI Exome Sequencing Project http://evs.gs.washington.edu/EVS/ supporting that these variants are not rare polymorphisms but disease causing mutations [20]. The patient carrying mutation G25V had no family history of arrhythmia. The patient carrying mutation G60D had an interesting phenotype of AF and borderline QT_c _interval that co-segregated from the mother to the son.

We used the *Xenopus laevis *oocyte system as it allows for better control of subunit ratios compared to transfection of mammalian cell lines such as CHO-K1. In *X. laevis *oocytes, KCNE1-G25V and KCNE1-G60D showed a gain-of-function for I_Ks _both with respect to steady-state current levels, kinetics, and heart rate-dependent modulation of I_Ks _(for G60D). When investigating the mutation in an experimental condition mimicking the heterozygous state of the patient, changes were not significant and hence the functional studies did not fully explain the phenotype, which could indicate that either the mutation is not causing the phenotype, or that the interaction is more complex. Yet, we showed gain-of-function effects for both identified mutations strengthening the notion that mutations in KCNE1 are associated with AF.

Furthermore, it has been shown that monoallelic expression is much more widespread than previously thought affecting 20% of human genes [21]. In a study addressing 190 genes on chromosome 21, *KCNE1 *was found to have 10 CG methylation sites rendering *KCNE1 *a profoundly epigenetically regulated gene [22] thereby making also the homozygote experimental condition highly relevant.

Though differences were less pronounced in mammalian cells, we still observed a gain-of-function of activation kinetics for G60D. Mutations leading to even mild gain-of-function of the I_Ks _current have been described earlier in the context of lone AF, yet all mutations identified so far reside in the α-subunit of the channel complex [18,23]. Two studies have investigated a possible association between AF and the SNP G38S in the β-subunit KCNE1 in Chinese AF cohorts. Lai et al. reported a significant association of 38 G, which was not found in the later study [24,25]. Of note, others observed decreased I_Ks _amplitudes and reduced surface expression with 38 G pointing to a loss-of-function of this variant [26].

The mutation G25V is located in the extracellular N-terminus of the channel protein. A recent study as implicated the extracellular juxtamembranous region of KCNE1 in gating [27], however, little is known about the role of the proximal KCNE1 N-terminus. The mutation G60D resides in the transmembrane segment (TMS) of KCNE1 in close proximity of residues 57-59 ("the gating triplet") that are critical for the modulation of K_V_7.1 channels [28]. Several glycine residues located in the TMS including G60 seem instrumental for forming a curvature that locates threonine 58 [29], which is critical for slow activation kinetics conferred by KCNE1 [30]. Hence, the mutation G60D in this close proximity could be speculated to compromise the function of this critical amino acid and thereby speed up activation [30-33].

Gain-of-function mutations of the I_Ks _channel are expected to increase the repolarising potassium currents which could abbreviate the cardiac action potential duration as well as the effective refractory period in cardiomyocytes. The mutation could thereby create a profibrillatory substrate within the atrium [10,11]. I_Ks _also plays a major role in ventricular repolarization. Gain-of-function of I_Ks _would be expected to result in both action potential and QT_c _shortening which we did not observe in the proband. It has been suggested that common variations in other genes may protect the patient from a shortening of QT_c _[31]. All mutation carriers in this study had not experienced ventricular arrhythmias, yet display a borderline long QT_c _interval. Pai and Rawles suggested a link between AF and prolongation of the mean QT interval [32]. The first study associating gain-of-function mutations in K_V_7.1 with AF analyzed a large four-generation family. Nine of 16 family members diagnosed with autosomal dominant hereditary AF showed prolonged QT_c _ranging from 450 to 530 ms [33]. Vice versa, Ackermann and colleagues reported presence of early-onset AF in a cohort of congenital LQT patients underlining the evidence for a link between these two syndromes [34]. Very recently, also a Brugada syndrome associated gain-of-function mutation in K_V_4.3, the α-subunit underlying the transient outward current I_to_, has been linked with QT_c _prolongation in the affected family member [35].

Despite the fact that proband 2 and his mother, both carrying the mutation G60D, had borderline long QT_c _intervals, there was no further suspicion of LQTS in the two patients. Clinical evaluation revealed no family history of syncope or near-syncope and the proband had a one year implantable loop recorder without any ventricular arrhythmias detected. Of note, both our index probands had an ECG pattern of IRBBB, which has recently been shown to be associated with early-onset lone AF [15] supporting the AF phenotype in the patients.

The exact mechanisms linking AF and QT prolongation and the different effect of I_Ks _mutations remain to be elucidated. One possible explanation may be different composition of the I_Ks _channel complex in atria and ventricles as suggested earlier to explain a mixed phenotype of AF and QT prolongation in a patient with a *KCNQ1 *mutation [18]. Also, chamber-specific interaction partners yet to be identified may modulate the effects of mutations in atria and ventricles. Furthermore, differences in triggers such as the well-documented beta-adrenergic stimulation [36] or the more recently described modulation by natriuretic peptide precursor A may be involved [23,37].

### Limitations

We limited our analysis to the *KCNE1 *encoding regions, and the possibility of mutations occurring in regions of the gene other than coding regions cannot be excluded. Though we performed genetic testing of all genes associated with AF earlier, we cannot exclude mutations in yet unknown genes. Furthermore, genetic testing of family members was limited as some were unavailable. Also, the number of probands was small, however, it should be noted that the cohort of young lone AF patients was well-defined. The functional analyses used conventional heterologous expression systems in which the environments differ from that in the native cardiomyocytes.

## Conclusions

In this study of lone AF patients, we found two suspected disease-causing mutations in *KCNE1*. Functional analysis of G25V and G60D showed a gain-of-function of the I_Ks _current. This study supports the hypothesis that gain-of-function in potassium current enhances AF susceptibility.

## Competing interests

The authors declare that they have no competing interests.

## Authors' contributions

MSO performed mutation screening, participated in the study design and wrote the manuscript. BHB participated in the study design, performed heterologous expression studies for G60D and the statistical analysis, and drafted the manuscript. JBN acquired clinical data and drafted the manuscript. ABS and JPD performed heterologous expression studies for G25V. JJ participated in the mutation screening. HKJ, SH and JHS acquired clinical data and helped to draft the manuscript. NS performed molecular biology and sequence alignment, conceived the study, participated in its design and coordination and wrote the manuscript. All authors read and approved the final manuscript.

## Pre-publication history

The pre-publication history for this paper can be accessed here:

http://www.biomedcentral.com/1471-2350/13/24/prepub

## Supplementary Material

Additional file 1 **The additional file (PDF) contains a detailed description of the methods, three additional figures (**Additional file 1**: Figure S1: Conservation of KCNE1 G25 and G60 within different species; **Additional file 1**: Figure S2: Comparison of I_Ks_-WT and I_Ks_-G25V channel currents in *Xenopus laevis *oocytes; **Additional file 1**: Figure S3: Comparison of I_Ks_-WT and I_Ks_-G60D and I_Ks_-WT/G60D channel currents.), and additional references**. Click here for file
