# Supplementary material for: Mutations in the potassium channel subunit KCNE1 are associated with early-onset familial atrial fibrillation
Source: BMC Med Genet. 2012 Apr 3;13:24. doi: 10.1186/1471-2350-13-24 (PMC3359244; doi:10.1186/1471-2350-13-24)
Supplement: Additional file 1 — The additional file (PDF) contains a detailed description of the methods, three additional figures (Additional file 1: Figure S1: Conservation of KCNE1 G25 and G60 within different species; Additional file 1: Figure S2: Comparison of IKs-WT and IKs-G25V channel currents in Xenopus laevis oocytes; Additional file 1: Figure S3: Comparison of IKs-WT and IKs-G60D and IKs-WT/G60D channel currents.), and additional references. [file 1471-2350-13-24-S1.PDF]

SUPPLEMENTAL INFORMATION

I. Detailed experimental methods

Expression in Xenopus laevis oocytes

X. laevis oocytes where purchased from EcoCyte Bioscience (Castrop-Rauxel, Germany). Oocytes were injected with $K_V7.1$ and KCNE1 WT or mutant in an 1:1 molar ratio (5 ng and 1 ng respectively) using a Nanoject microinjector (Drummond, Broomell, PA, USA). Oocytes were kept in Kulori solution (in mM: 87 NaCl, 4 KCl, 1 $MgCl_2$, 1 $CaCl_2$, 5 HEPES, pH 7.4) at 19°C. Experiments were performed 3 days after injection.

Two-electrode voltage-clamp

Two-electrode voltage-clamp (TEVC) experiments were performed using a Dagan CA-1B amplifier (Dagan Corporation, Minneapolis, MN, USA). Oocytes were placed in a perfusion chamber under continuous superfusion with Kulori solution at 21-23°C. Glass pipettes for recording electrodes were pulled from borosilicate glass capillaries (Module Ohm, Herlev, Denmark) on a DMZ Universal Puller (Zeitz Instruments, Munich, Germany). When filled with 2 M KCl the tip resistance was between 0.5 and 2.5 MΩ. Data acquisition was performed with the Pulse software (HEKA Elektronik, Lambrecht/Pfalz, Germany). For all recordings, the holding potential was -80 mV.

Cell culture and patch clamp experiments

Experiments were performed with an EPC-9 amplifier (HEKA), and recordings were low-pass filtered at 3 kHz and acquired using PULSE software (HEKA). Pipettes were pulled from borosilicate glass (Module Ohm) with a resistance of 1.5-2.5 MΏ.

Chinese Hamster Ovary (CHO)-K1 cells were cultured in Dulbecco´s Modified Eagle Medium (DMEM) (Life Technologies, USA) supplemented with 10% fetal calf serum (Life Technologies) and grown at 37°C in 5 % CO_2. Cells were transfected with $K_V7.1$ (1 µg) and KCNE1 or KCNE1-G60D (0.2 µg) cDNA using Lipofectamine (Life Technologies, USA). Two days after transfection cells were trypsinized and transferred to coverslips for electrophysiological experiments. The extracellular solution used consisted of (in mM) 140 NaCl, 4 KCl, 2 $CaCl_2$, 1 $MgCl_2$, and 10 HEPES, pH 7.4 and was heated to at 36±1°C using an

in-line solution heater and an automated temperature controller (Warner Instruments, Hamden, CT, USA). The intracellular solution used for whole cell measurements consisted of (in mM): 140 KCl, 10 EGTA and 10 HEPES pH 7.2. $CaCl_2$ and $MgCl_2$ were added in concentrations calculated (EqCal; BioSoft, Cambridge, UK) to give a free Ca^{2+} concentration of 100 nM and a free Mg^{2+} concentration of 1 mM. For whole cell recordings, cell capacitance and series resistance were updated before each pulse application. Series resistance values were between 2.5 and 8.0 MΩ and only experiments where the resistance remained constant during the experiments were analyzed.

II. Supplemental Data

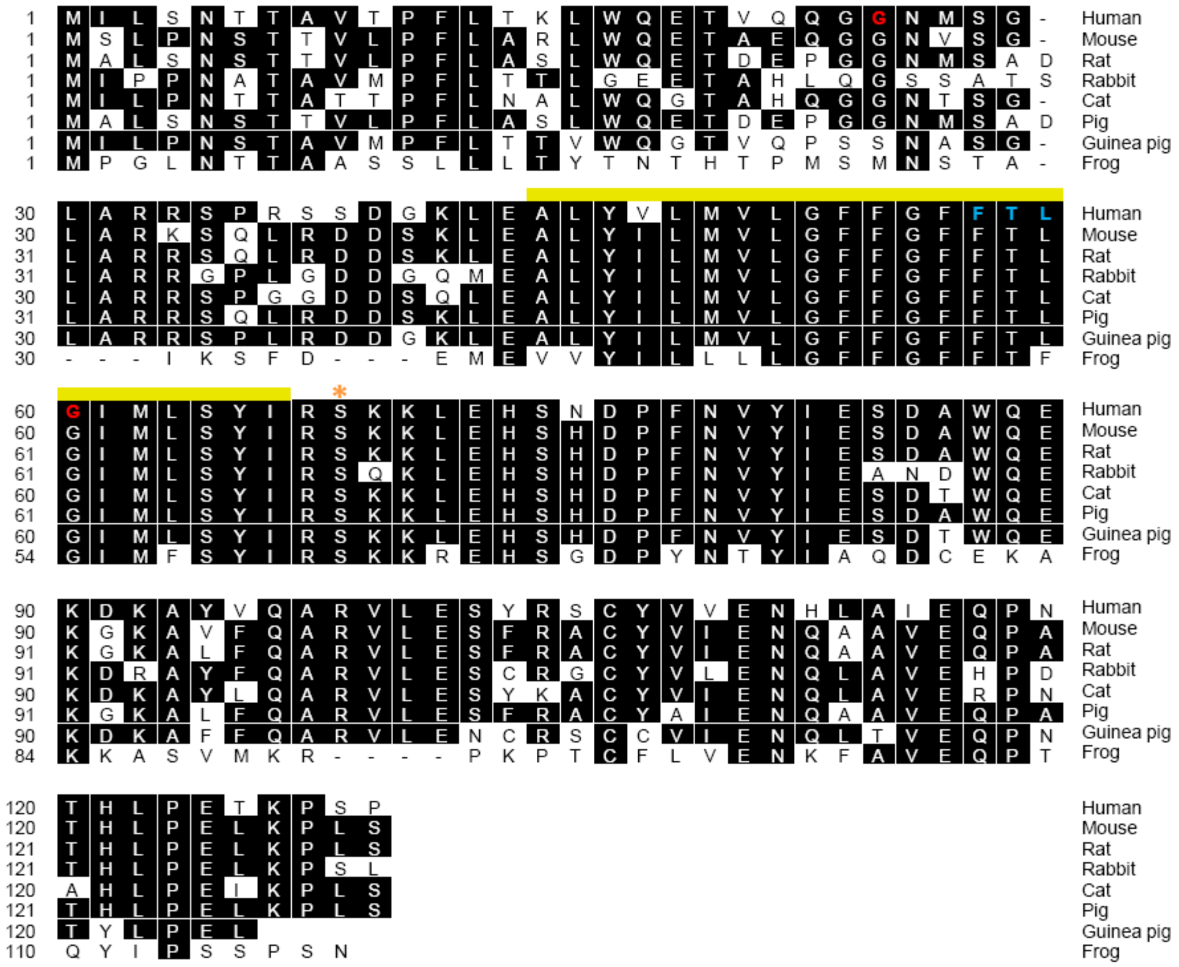

Online Figure 1: Conservation of KCNE1 G25 and G60 within different species. Amino acid alignment of KCNE1 sequences (GenBank Acc. No. in brackets) from cat (Q9XSP1), frog (AAN77244), guinea pig (Q60409), human (NP_000210), mouse (NP_032450), pig (BAA86982), rabbit (Q28705), and rat (NP_037105) using ClustalW with default settings. Marked in red are the residues G25 and G60 investigated in this study. The yellow bar above the alignment depicts the putative transmembrane region; the orange star marks the conserved PCK phosphorylation site. Blue letters denote the "activation triplet" found to determine gating specificity[1]

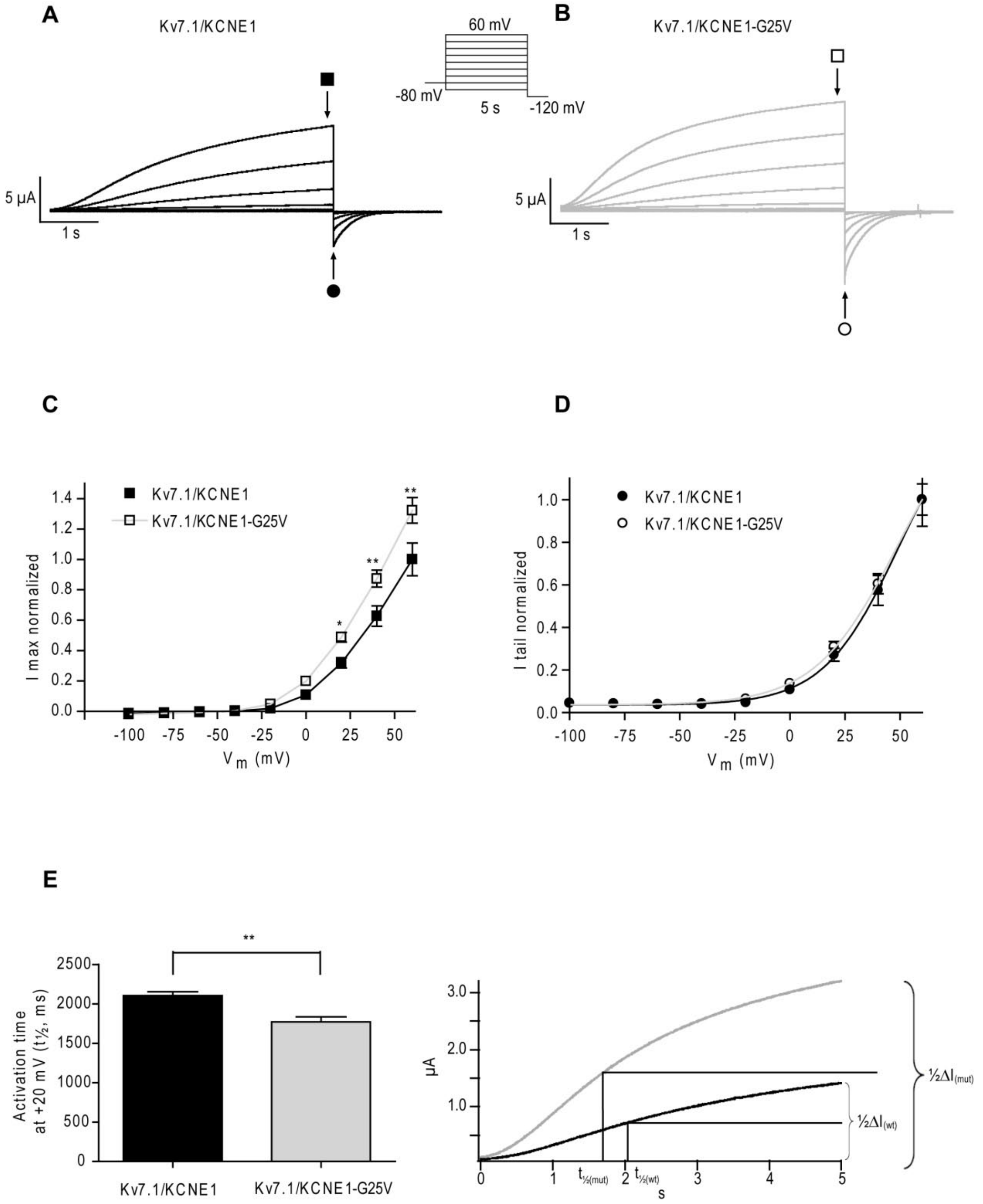

Online Figure 2: Comparison of I_{Ks}-WT and I_{Ks}-G25V channel currents. Representative current traces for KCNE1-WT (**A**) and KCNE1-G25V (**B**) channel subunits co-expressed with K_V7.1 in *X.laevis* oocytes in a 1:1 molar ratio. Current step protocol is shown as inset.

C: I/V relationship for I_{Ks}-WT (n=15) and I_{Ks}-G25V (n=22) as determined from peak currents (open and filled squares). **D**: Voltage-dependence of I_{Ks}-WT and I_{Ks}-G25V channel activation as determined from tail currents (open and filled circles). **E**: Activation rise time, determined as the time to ½ max following a depolarization to +20 mV.

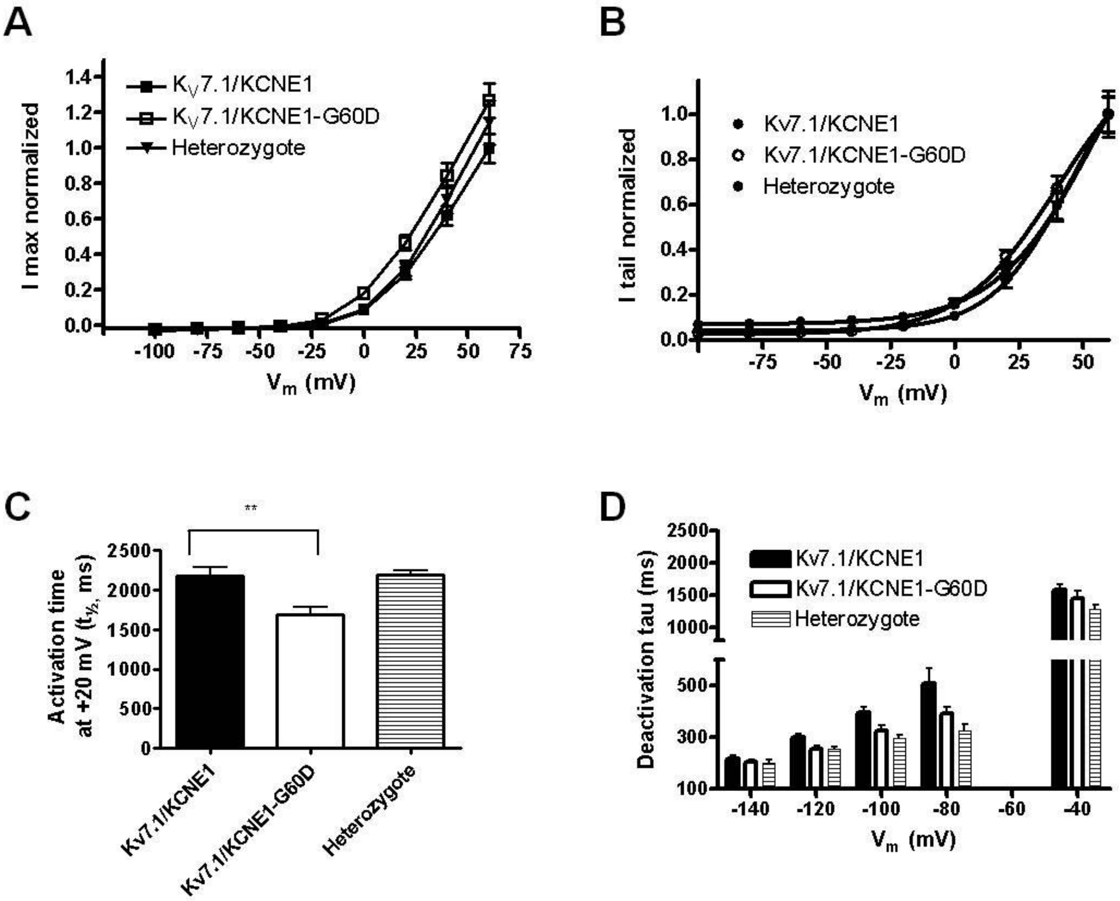

Online Figure 3: Comparison of I_{Ks}-WT and I_{Ks}-G60D and I_{Ks}-WT/G60D channel currents. KCNE1-WT, KCNE1-G60D and KCNE1-WT/G60D channel subunits co-expressed with K_V7.1 in *Xenopus laevis* oocytes in a 1:1 molar ratio. Currents were elicited as explained in figure 2 and 4. **A**: I/V relationship for I_{Ks}-WT (n = 21), I_{Ks}-G60D (n =23) I_{Ks}-WT/G60D (n=16). Currents were measured at the end of the 5-second test pulse and normalized to the maximum current in K_V7.1/KCNE1. **B**: Voltage dependence of channel activation. Peak tail currents were measured at -120 mV, and the normalized data were fit to a

two-state Boltzmann distribution. **C**: Activation rise time, determined as the time to ½ max following a depolarization to +20 mV. **D:** Deactivation time constants (tau) were obtained by fitting the tail current traces to a mono-exponential function.

III Supplemental References

1. Melman YF, Domènech A, de la Luna S, McDonald TV. Structural determinants of KvLQT1 control by the KCNE family of proteins. *J. Biol. Chem* 2001;**276**:6439-6444.
